# Correlations between cresty neck scores and post-mortem nape fat measurements in horses, obtained after photographic image analysis

**DOI:** 10.1186/s13028-016-0241-4

**Published:** 2016-10-20

**Authors:** Severiano R. Silva, Rita Payan-Carreira, Cristina M. Guedes, Simão Coelho, Ana Sofia Santos

**Affiliations:** 1 Zootecnia Department, Centro de Ciência Animal E Veterinária, Universidade de Trás-os-Montes e Alto Douro, 5000-801 Vila Real, Portugal; 2 EUVG-Escola Universitária Vasco da Gama, Campus Universitário, Bloco B, Lordemão, 3020-210 Coimbra, Portugal; 3 CITAB, Universidade de Trás-os-Montes e Alto Douro, Quinta de Prados, 5000-801 Vila Real, Portugal

**Keywords:** Adiposity, Cresty neck score, Image analysis, Body condition, Obesity, Horses

## Abstract

**Background:**

Obesity and emaciation in horses have major detrimental effects on health and morbidity, reproductive failure, work performance or carcass quality. Scoring is a current management tool used to assess and monitor equine body condition due to its simplicity and low cost. However, accurate assessment of obesity remains a challenge, even though a number of approaches have been tested, particularly for research purposes on adiposity. Their merit is usually validated by comparison with standard scoring methods. The overall aim of this study was to establish the correlation between post-mortem nape fat measurements obtained after photographic image analysis and cresty neck score (CNS) in horses. Data were collected from seventeen horses with a hot carcass weight of 165 ± 51 kg. Pre-slaughter CNS measurements were obtained using a six-point scale (from 0 to 5). Image capture was performed post-mortem, in the slaughter line; for each carcass, images of the dorsal and medial views were collected and afterwards transferred to a computer for analysis. After outlining the cresty neck fat, its area, major axis and thickness were determined. Correlation coefficients between nape fat measurements, CNS and carcass fatness were determined.

**Results:**

The horses in the study show similar variation for CNS and hot carcass weight [Coefficient of variation (CV) = 32 and 31 %, respectively], but a high variation for carcass fattening (CV = 41 %). The nape fat area measurement was the parameter exhibiting the greatest variation (CV = 50 %). Correlations established between CNS and the variables tested revealed the existence of moderate to strong correlations among CNS, nape fat measurements, and carcass fatness. The highest correlation coefficients were found between CNS and nape fat thickness (r = 0.882; P < 0.01). The linear regression between CNS and nape fat thickness accounted for 77 % of the recorded variation for nape fat thickness.

**Conclusions:**

The present study showed that there is a strong correlation between horse CNS and post-mortem nape fat measurements or carcass fatness.

## Background

Obesity and emaciation pose significant problems in horses and have been associated with an increased risk of health and morbidity, reproductive failure, and poor work performance or carcass quality [1–3]. The ability to quantify and monitor animal body condition is a key issue for successful equine production and may also contribute to animal welfare [4, 5].

Body condition scoring (BCS) is currently the most common management tool for assessing and monitoring body condition in horses. Henneke’s nine-point scale [6] is probably the most widely used scoring system, outlining the analysis of fat deposition over diverse body regions. Recently, a new 5-point scoring system was developed, focused on grading neck crest fatness [7]. Increased adiposity in specific locations, namely the fat deposits along the crest of the neck, has been associated with disequilibrium in the metabolic state and with an increased risk of metabolic disorders and inflammatory states [8]. Although body condition scoring (BCS) systems are useful to assess equine obesity, cresty neck scores (CNS) are a better predictor of the risk of metabolic diseases [8]. Therefore, CNS may be highly beneficial in screening levels of neck crest adiposity and recognising animals at an increased risk of obesity-related diseases [9].

Developed by experienced observers, scoring methods depend on a subjective score supported by a range of classified anatomical descriptors focused on a horse’s external form/frame [6, 7]. These scoring systems are currently applied to horses and ponies due to their simplicity and reduced cost, although they require the operator to be experienced in judging condition as well as sufficiently practised in obtaining consistency of scores. More objective morphometric measurements (such as the girth-height ratio or the neck circumference) are, nonetheless, available to assess adiposity in particular body regions [7, 10, 11]. However, assessing obesity remains a challenge, due both to under-recognition of obesity by owners and the limitations in available scoring and measurement systems [11].

In an attempt to avoid these constraints, in particular for research purposes on horse adiposity, several methods and techniques have been tested to determine body condition in horses. These include tracer dilution methodologies, such as deuterium oxide [12], bioelectrical impedance analysis [13] or image techniques, which include real-time ultrasonography [14–16]. The merits of all these techniques are usually validated by comparison to matched BCS [6] or CNS [7], which have recognised limitations, including the influence of breed or body size on scoring measurements or lack of knowledge about their relationship with the actual content of body fat [17]. CNS being a subjective method, small variations may remain undetected even if good inter-observer reliability has been established [8].

Although CNS is a commonly used method to assess nuchal crest adiposity, it has not been formally validated using post-mortem methods, as recognised by Giles et al. [8]. Therefore, the overall aim of this study was to establish the correlation between nape fat measurements obtained in carcass after photographic image analysis and CNS in horses.

## Methods

### Animals

Data were collected in a national abattoir from 17 horses aged 3–9 years old, with a hot carcass weight of 165 ± 51 kg. No account was taken of nutritional history or of the breed of the horses. All horses slaughtered were approved for meat consumption; only animals with non-existing metabolic disease or signs of trauma (according to sanitary inspection documents) were used. For data collection prior to slaughter, animals were handled in accordance with International Ethical standards. The official entities regulating meat inspection and the management of the industrial facilities gave their permission to conduct the study within the abattoir and for data collection on carcasses, as required by national laws.

### Collection of data

Cresty neck scores measurements were obtained before slaughter by two skilled evaluators (SC and SRS) while animals were in the lairage. CNS was assigned on a six-point scale (from 0—no palpable crest to 5—enormous crest), with half-score increments, according to the system described by Carter et al. [7].

**Fig. 1:**
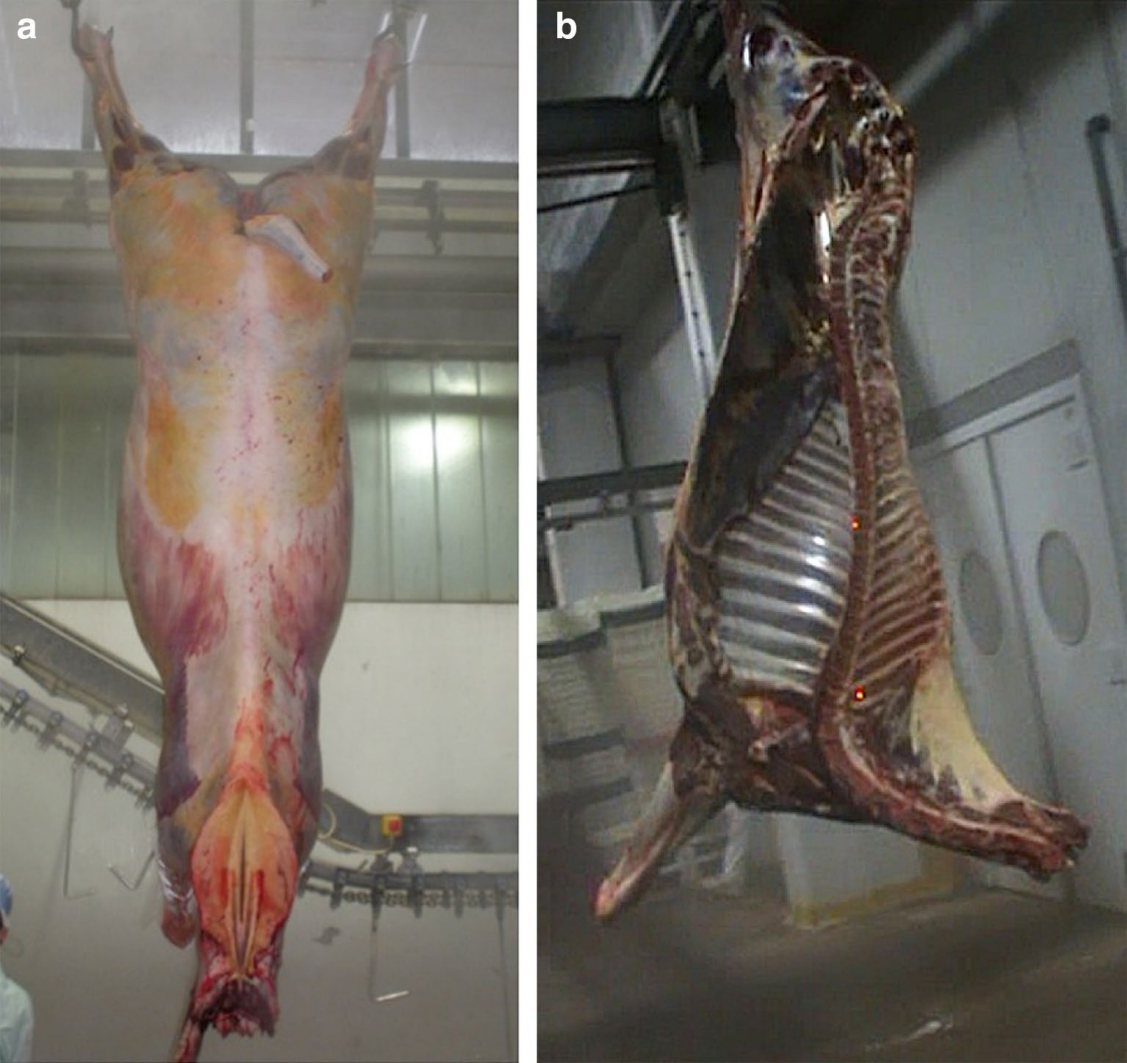
Collection of images at the abattoir. Example of the dorsal side (**a**) and the medial side (**b**) images taken at the slaughter-line

**Fig. 2:**
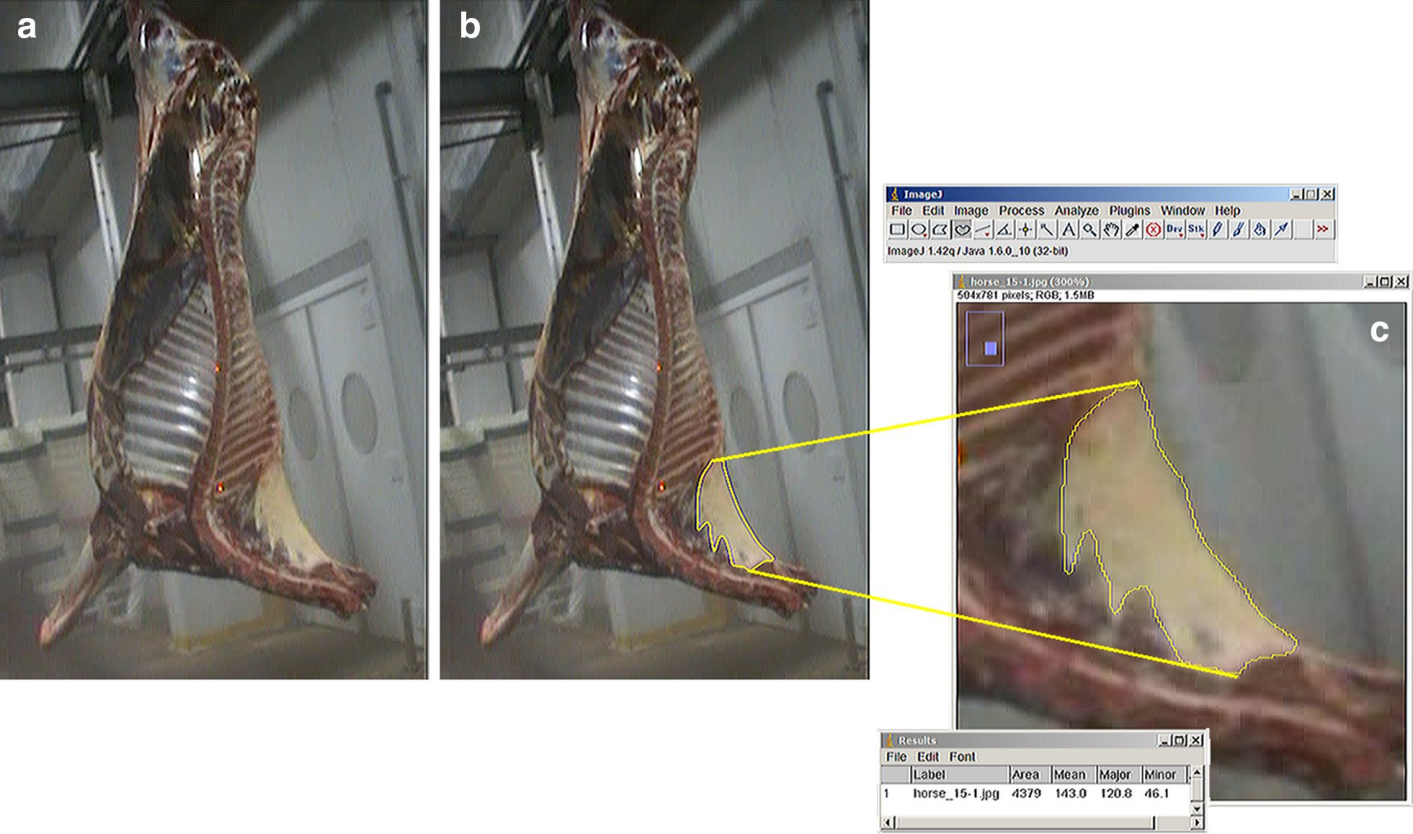
Nape fat evaluation in carcasses at the abattoir. A medial view of hemi-carcass showing the two red laser points used as scale (**a**); outline of the nape fat using the *ligamentum nuchae* as reference (**b**) and a detail of ImageJ application to obtain the nape fat measurements (i.e., area, major axis and the minor axis (thickness of the nape fat) (**c**)

The capture of carcass images was performed in an efficient manner so as to avoid any disturbances to workflow in the slaughter line. Images of dorsal and medial views were collected for each carcass (Fig. 1). Images representing the dorsal nuchal view were taken before the longitudinal section of the carcass, whereas the images corresponding to the medial side view were captured in the resulting hemi-carcasses, before weighing. Care was taken to completely immobilize the (hemi-) carcasses before capturing images. Images were captured using a digital camera (Nikon Coolpix S210) featuring an 8 Megapixel sensor. The camera was set as follows: shutter speed 1/10 s; manual operation mode; aperture Av F/4.6; ISO velocity 200; flash off; focal distance 74 mm; f 3.3. Captured images were saved as 3264 × 2448 pixel JPEG format photographs. The camera was placed 4 m from the carcasses, under standard artificial light. In addition, during the capture of images from the medial view of hemi-carcasses, two red laser dots were projected on the carcass (Fig. 2a), for scale-bar purposes; the two red lasers, each with a 650 nm wavelength, were mounted on a frame, held in a position parallel to the shot area, with a predetermined distance between the two lasers of 32 cm.

Carcass fatness was graded using an adapted 15 point scale described by Bohuslávek [17], derived from fat cover classification developed for adult bovine carcasses, and proposed for horses by Sarriés and Beriain [18]. Grading of carcass fatness was performed from its dorsal view, by a researcher (SC).

### Image analysis and nape fat measurements

Captured images were transferred to a computer for analysis and a new identity code was randomly attributed, for blind assessment. A single operator performed the image analysis, using ImageJ software (version 1.38×, NIH, USA). To perform the measurements, the neck crest fat was outlined by means of ImageJ’s freehand area tool, using the nuchae ligament as an anatomical reference point (Fig. 2b, c). The major axis and the minor axis, representing the thickness of nape fat, were automatically determined after setting the area measurement selection (Fig. 2c). The distance between the two red laser dots (32 cm) was used in ImageJ to give pixels real dimension; the result in pixels was entered in the software’s set scale box.

### Statistical analyses

All statistical tests were analysed using JMP software (version 7, SAS Institute, Cary, NC, USA). After testing data normality, simple descriptive statistical analyses were performed. Results are presented herein as mean ± standard deviation, minimum and maximum values and coefficient of variation (CV). The correlation coefficient (r) between nape fat measurements, CNS, and carcass fatness were determined after correlation analysis. A linear regression between the CNS scores and the thickness of nape fat was also performed. The coefficient of determination (R^2^) was used to evaluate the ability of the prediction equation.

## Results

Table 1 summarises the results for hot carcass weight, CNS, fat carcass level and nape fat measurements of the horses studied.

**Table 1:** Hot carcass weight, cresty neck scores (CNS), carcass fat and nape fat measured in horses (n = 17)

Parameter	Mean	SD	Min.	Max	CV (%)
Hot carcass weight, kg	164.6	50.7	105	272	30.8
CNS, 1–5	2.35	0.745	1.00	3.00	31.7
Carcass fat level, 1–15	7.24	2.93	3.00	13.0	40.5
Nape fat measurements					
Major axis (cm)	17.1	4.34	11.0	26.3	25.3
Area (cm^2^)	310.5	156.3	120.5	691.8	50.3
Thickness (cm)	8.99	3.07	3.30	14.2	34.1

*SD* standard deviation; *Min.* minimum values; *Max.* maximum values; *CV* coefficient of variation

The 17 horses showed similar variation for CNS and hot carcass weight (CV of 32 and 31 %, for CNS and hot carcass, respectively; Table 1). However, they exhibited greater variation with respect to carcass fattening than CNS (CV = 41 vs. 32 %, for carcass fattening and CNS, respectively). Interestingly, the nape fat area measurement was the parameter showing the greatest variation (CV = 50 %), while the other two nape fat measurements revealed a CV of 25 and 34 % for major axis and nape fat thickness, respectively, which was in line with the variation observed in CNS.

The correlations established between the CNS and the other variables tested in the present study are presented in Table 2. The data showed that moderate to strong correlations existed among CNS, nape fat measurements, and carcass fatness. There were strong correlations between CNS and nape fat variables, with higher correlation coefficients within nape fat thickness (r = 0.882; P < 0.01). The correlation coefficients were similar between CNS and the other nape fat variables (r = 0.737 and r = 0.691 for the nape fat major axis and area, respectively; and 0.760 for carcass fatness; P < 0.01). Additionally, a strong correlation also existed between the various measurements taken for nape fat (r between 0.827 and 0.991, P < 0.01).

**Table 2:** Correlation among cresty neck scores (CNS), nape fat measurements and carcass fatness in horses (n = 17)

Nape fat measurements	Major axis	Area	Thickness	Carcass fatness
CNS	0.737	0.691	0.882	0.760
Major axis		0.991	0.840	0.596
Area			0.827	0.558
Thickness				0.734

All correlation coefficients are significantly (P < 0.01) different from zero

**Fig. 3:**
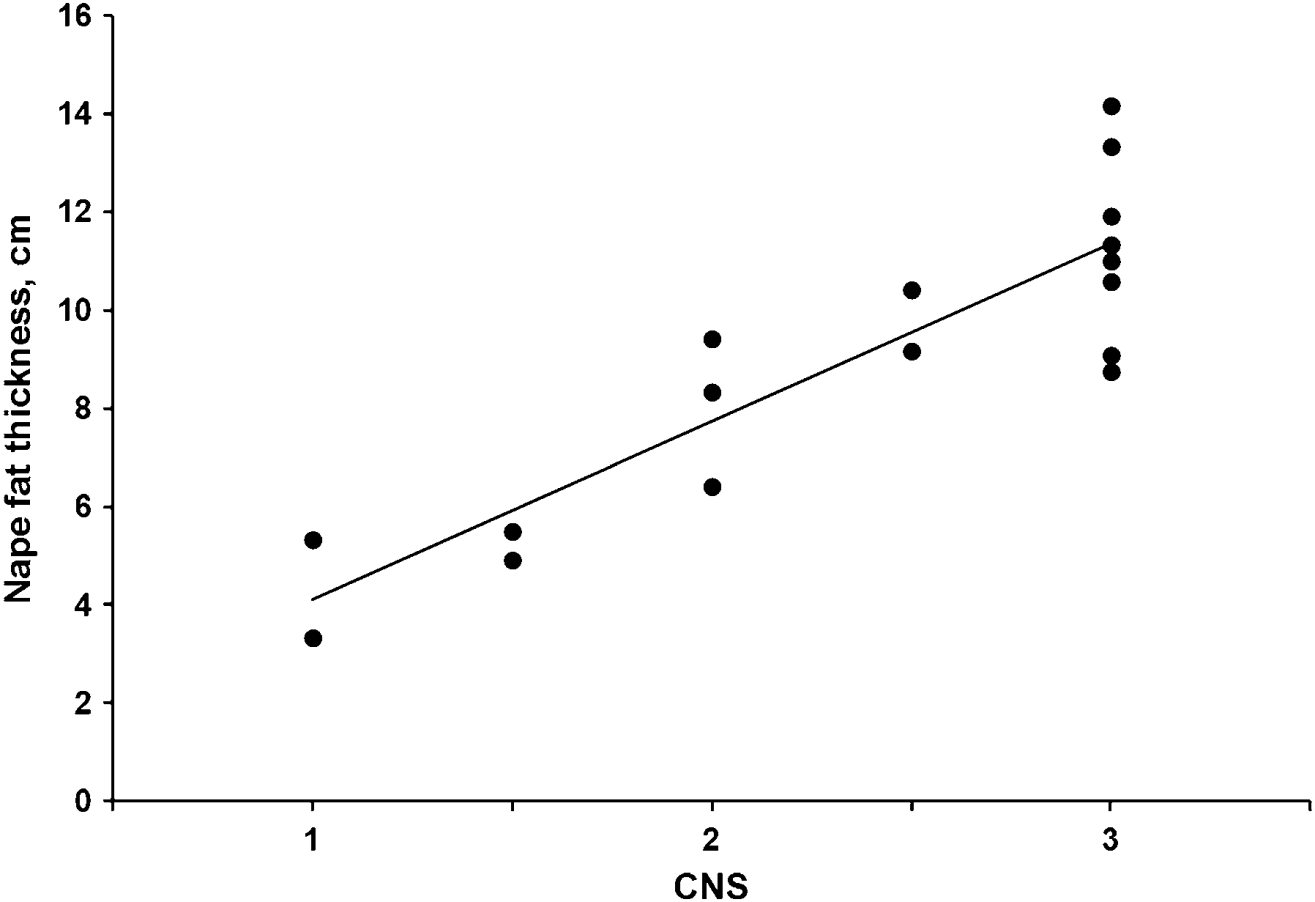
Linear relationship (y = 3.612× + 0.5011; R^2^ = 0.774) of nape fat thickness and cresty neck score (CNS) in horses

**Fig. 4:**
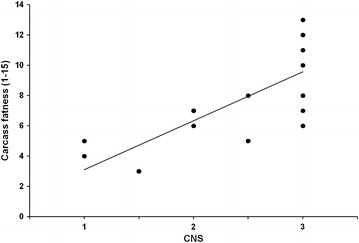
Linear relationship (y = 3.229×–0.1258; R^2^ = 0.578) of carcass fatness and cresty neck score (CNS) in horses

The linear regressions explaining the relationship between CNS and nape fat thickness or CNS and carcass fatness are presented in Figs. 3, 4, respectively. CNS explains 77 and 58 % of the observed variation of nape fat thickness and carcass fatness, respectively.

## Discussion

This study details the relationship between CNS and matching nape fat measurements. Typically, to establish a relationship between fat scores (e.g. CNS) and other body composition traits, the use of the widest range of variables is sought. In the present study, CNS ranges from 1 to 3, with no values of 0 and 0.5 or between 3.5 and 5. Nevertheless, the variation of the fattening traits assessed here (CV between 25 and 50 %) is comparable to that presented in other studies using horses with a carcass weight range of 105–418 kg [14, 19–21]. These studies showed that fat was the most variable component of the body (CV between 18 and 43 %) [14, 20] and in carcasses (CV between 33 and 73 %) [19, 21]. More recently, Verhees [22] confirmed that horses displaying a CV of 22 % for live weight showed higher variations for CNS and BCS (CV 45 vs. 38 %, for CNS and BCS, respectively).

Results from the present study also demonstrated the existence of a significant correlation between CNS and fat measurements or carcass fatness. This is an interesting result that may allow further research to address issues regarding adiposity and adiposity variation in horses. Giles et al. [23] recently showed that seasonal changes of neck crest adiposity differ from those of overall adiposity, suggesting that the importance of adiposity may differ in different regions, even if both parameters are commonly associated. Carter et al. [7] point out two main limitations for CNS: the fact that it only assesses visibly apparent or appreciable neck crest adiposity, and the lack of breed or size references for horses. Therefore, CNS would only really be useful when used as a ratio to neck length [23]. However, the use of image-based neck crest parameters may attract additional interest, particularly for research on horse nutrition, adiposity, and metabolic disease risk factors. Further studies should seek to address the relation between nape fat measurements and nape fat thickness and features as measured by ultrasound in a larger cohort of animals, covering a wider range of CNS in different periods of the year. Recent studies by Giles et al. [8] report the existence of an apparently opposite pattern in the seasonal variation of CNS, compared to general obesity in domestic horses and ponies: CNS values were higher in winter compared to summer. Whether or not this difference reflects a real winter increase in regional fat deposition or whether it results in differences in the risk of metabolic stress still needs to be ascertained.

The present study also establishes a strong correlation between CNS and carcass fatness, which is supported by findings from other authors, who have found a strong relationship between nape fat and body or carcass adiposity [21, 24], and it supports a broader understanding of the mechanism of body adiposity in horses through the use of indicators based on nape fat.

Despite the lack of breed or size references, regarded by Giles et al. [23] as a significant pitfall for CNS in practice, Morrison et al. [24] argue that the strong relationship between nape fat and BCS is not confounded by an animal’s phenotype. It is noteworthy that the present study revealed that nape fat thickness showed the best correlation with CNS.

## Conclusions

Our results have revealed the existence of a significant correlation between CNS and nape fat measurements in horse obtained after image analysis, in particular with nape fat thickness. The present study has, moreover, established a strong correlation between CNS and carcass fatness. In future research, a sample with a wider range of body weights and sizes would be beneficial to get a better understanding of the relationship between CNS and carcass fatness in horses.

## Abbreviations

BCS: body condition scoring
CNS: cresty neck scores
CV: coefficient of variation
r: coefficient of correlation
R^2^: coefficient of determination
